# Integrated Optic Sensing Spectrometer: Concept and Design

**DOI:** 10.3390/s19051018

**Published:** 2019-02-27

**Authors:** Gloria Micó, Bernardo Gargallo, Daniel Pastor, Pascual Muñoz

**Affiliations:** 1 Photonics Research Labs, Universitat Politècnica de València, c/ Camino de Vera s/n, 46021 Valencia, Spain; glomica@iteam.upv.es (G.M.); dpastor@dcom.upv.es (D.P.); 2 VLC Photonics S.L., c/ Camino de Vera s/n, 46021 Valencia, Spain; bernardo.gargallo@vlcphotonics.com

**Keywords:** integrated optical sensor, evanescent-field sensing, absorption spectroscopy

## Abstract

In this paper the concept and design of an integrated optical device featuring evanescent field sensing and spectrometric analysis is presented. The device, termed integrated optics sensing spectrometer (IOSS), consists of a modified arrayed waveguide grating (AWG) which arms are engineered into two sets having different focal points. Half of the arms are exposed to the outer media, while the other half are left isolated, thus the device can provide both sensing and reference spectra. Two reference designs are provided for the visible and near-infrared wavelengths, aimed at the determination of the concentration of known solutes through absorption spectroscopy.

## 1. Introduction

Photonics is a key enabling transversal technology, serving multiple application domains, from telecom/datacom, through to bio/life sciences, avionics/aeronautics, safety, security to civil engineering and construction [1]. As part of this field, photonic integrated circuits (PICs) are becoming ubiquitous and three mainstream integration technologies, namely silicon-on-insulator (SOI) [2], indium phosphide (InP) [3] and silicon nitride (SiN) [4] have been established. Selecting one or other technology for a PIC is tightly matted to the application and operation wavelength range [5].

Among the different PIC applications, photonic on-chip sensors have been the subjects of study since the early 1980’s [6] and the advance in these three decades lead to an state of the art on which integrated optics structures (waveguides, slot waveguides, gratings, etc.) have been applied to sense, mainly using evanescent fields, in different application domains [7]. These structures have been traditionally incorporated into relatively simple (from a telecommunication/signal processing perspective) interferometric devices, being the Mach–Zehnder interferometer (MZI) [7] and ring-resonators (RR) [8] (cavities in general) being the most used. The relative simplicity of these devices, yet not the science of making them sensitive to the analytes (i.e., functionalization for specificity), allows a neat transduction of the sensed magnitude into an optical and/or electrical measurable signal. However, a significant amount of complexity resides in the readout system. In a considerable number of cases, an optical spectrum is acquired, since induced changes in the on-chip optical signals are reflected in the power spectral density shape. Acquisition is done with costly off-chip bulky instruments (as tunable lasers with photodetectors, or broadband sources with optical spectrum analyzers). Hence, bringing a spectrometer into the chip area will suppose a significant progress over the current state of the art of photonic chip sensors, aligned with wearable chip sensors technologies [9].

**Figure 1:**
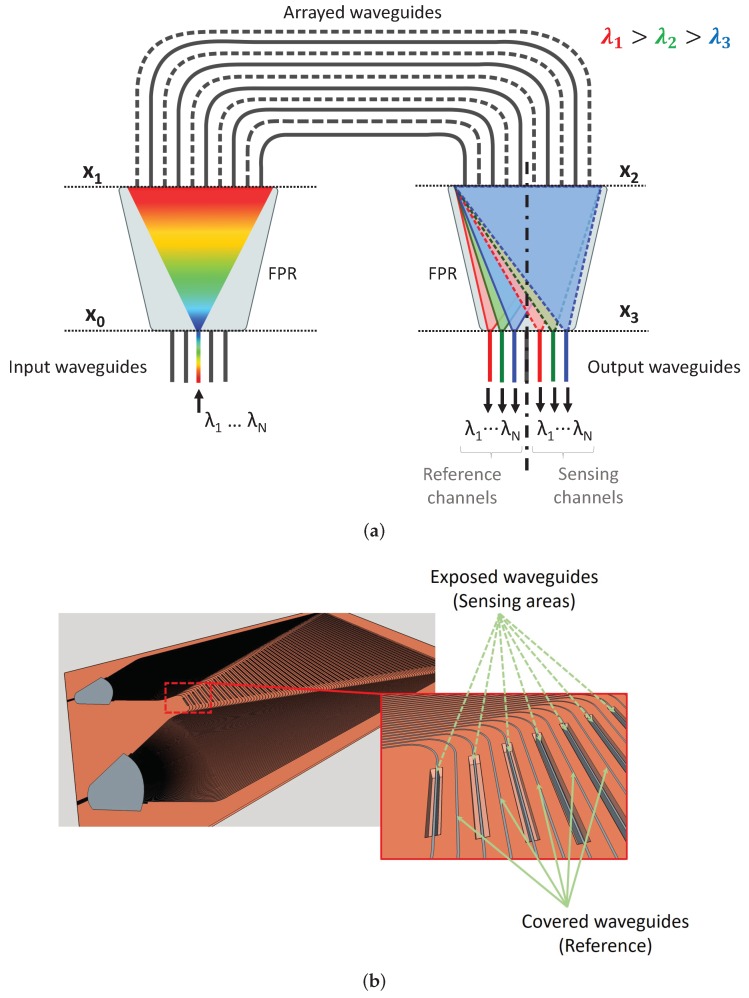
(**a**) Integrated optics sensing spectrometer (IOSS) operation scheme. (**b**) IOSS layout for frequency shift analysis. Windows are opened (exposed waveguides) in one of the sub-arrays of the waveguide grating (AWG) to interact with the analyte while the other sub-array remains covered.

Whereas integrated optics wavelength filtering for telecom has achieved strong maturity, conventional spectrometric techniques usually employ bulk, heavy and large (commercial) instruments with tuning and reconfiguration capabilities, such as mechanically actuated parts with grating plates and varying slit widths. Some commercial miniaturized spectrometers that make use of two face to face chips have been developed on a microelectromechanical systems (MEMS) fabrication process, but they are still based on free space optics principles [10,11]. For waveguide integrated spectrometers, among others, there are two building blocks used in PICs: the Echelle diffraciton grating (EDG) [12] and the arrayed waveguide grating (AWG) [13]. A conventional AWG consist of two free propagation regions (FPR, zone where the field is diffracted along the horizontal direction) interconnected by a plurality of waveguides, commonly called arrayed waveguides (Figure 1a). There is a length incremental between consecutive waveguides, ΔL, which determines a phase shift depending on the wavelength applied. At the output plane (plane $x_{3}$, Figure 1a), the contributions of all the arrayed waveguides are added together. Those positions where light constructively interfere for different wavelengths are known as AWG focal points, and that is where the output waveguides are placed to collect the light.

In contrast, an EDG is a grating mirror with step-shaped grooves with narrow facets in which the incident light is diffracted back, so only one FPR is required. Since the angle of diffraction depends on the wavelength, the demultiplexation of the signal is achived. For some specifications, the EDG is more compact. Nonetheless, the AWG offers further degrees of freedom for design and reconfiguration [14]. Photonic sensing applications do often resort to wavelengths other than the optical telecom C-band (1530–1565 nm), because of which some groups have demonstrated static AWG spectrometers going from the visible (620 nm) [15] up to long near-infrared wavelengths (5 μm) [16].

In this paper we present a device concept that combines in the core of a single device evanescent field sensing and spectral analysis. The device is termed integrated optics sensing spectrometer (IOSS) and builds upon a device previously proposed by C. Doerr, the interleave-chirp AWG (IC-AWG) for coherent optical communications [17,18,19]. Optical spectrometers are usually employed as optical sensors to obtain the Raman or absorption spectrum of a sample, as declared in [20]. Several examples can be found for Raman spectroscopy [21] and absorption spectroscopy [15,22,23] applications. All the devices shown in these examples work with liquid samples as IOSS does. In the case of absorption spectroscopy applications, the sensor for glucose analysis presented in [23] employs a broadband source and a tunable filter to perform the spectroscopic study. On the contrary, devices presented in [15,22] take advantage of the intrinsic demultiplexing feature of AWG to avoid the use of bulky instrumentation to realize the analysis. All the devices mentioned so far have the same characteristic; in all of them the sensing and spectroscopy analysis is performed in separated areas. This implies the need of using several components within the sensor to achieve absorption spectrometry, therefore the footprint of the device is increased. Based on these data, IOSS device possess two clear advantages over the existing spectroscopic devices. First, reference and sensing measurements are conducted simultaneously, therefore the ambient conditions do not affect the results. Relative measurements between the reference and sensing channels are always taken as the final result, as done in [23]. Second, sensing and spectral measurements are performed in a single device so the complexity and size of the PIC are reduced.

This paper is structured as follows: the device concept is presented in Section 2, followed by the minimal set of equations to describe its behavior and design in Section 3. Reference designs are provided in Section 4, where insight is given in several aspects of the resulting devices. Finally, the conclusion and outlook are presented in Section 5.

## 2. Device Concept

A sketch of the device is presented in Figure 1. The IOSS is an AWG where half of the arrayed waveguides, referred to as arms, are employed for sensing (sensing sub-array), whereas the other half are used to provide the reference for the input signal (reference sub-array), as shown in Figure 1a with dashed and solid lines respectively. The two sub-arrays are interleaved as in [17,18,19]. The arms in the sensing sub-array are equipped with sensing windows, Figure 1b, which in an integrated optics technology can be attained by selectively etching away the cladding. This allows for the analyte under study to interact with the evanescent optical field in the waveguides. The interaction between the analyte and the optical field may change the propagation conditions, either by altering the real part (phase change), the imaginary part (absorption change) of the effective refractive index, or both. To be able to spatially separate the optical signals from the sensing and reference sub-array, each of the two sub-arrays is designed to have a different focal point, i.e., output spatial coordinate of the AWG, for a given wavelength (see $x_{3}$ plane in Figure 1a). In an AWG, this is controlled by the length increment between consecutive waveguides, ΔL [24]. Hence, the output plane is divided into two halves, each one independently collecting the optical signals coming from the sensing and reference sub-arrays.

Since only one of the sub-arrays is equipped with sensing windows, only the properties of the corresponding focal points (sensing focal points) will be altered, while the reference focal points will remain unperturbed. The relative change between the reference and sensing focal points can be correlated with the presence and nature of a given analyte, through a transduction method. The transduction method employed relates the changes in the signal with certain physical or chemical properties of the sample. For example, monitoring refractive index changes (frequency shift) or fitting the absorption spectra may provide information about the concentration of the analyte. By enabling multiple reference and sensing focal points for different wavelengths, the wavelength dependence (spectroscopy) of the light–analyte interaction can be recorded. Hence, in a single step, both reference (background) and analyte spectra can be obtained.

The IOSS design will change depending on the transduction method employed. For absorption analysis, all the sensing windows are set to have the same length, so as all the paths experience the same amount of attenuation due to the analyte. Furthermore, having all windows the same length, there is no relative phase change among arms due to the analyte, and hence no displacement of the focal points at the output plane ($x_{3}$) happens. On the contrary, for frequency shift analysis, the sensing windows must have a constant incremental length, Figure 1b, between adjacent sensing windows to ensure the focal displacement.

## 3. Device Design

In this section, the design procedure is presented in a technology agnostic way, and some reference design will be provided later on. The design procedure stated here pretends to show the main steps differing from a conventional AWG design, which can be found in our previous works on AWG modelling and design [14] and also in Interleaved Chirped-AWG [19].

Firstly, the integration technology selected, together with the frequency chosen for our application dictated the fundamental waveguiding parameters:

- $\nu_{0}$, referred as central frequency (bandpass frequency between a pair of input and output waveguides),
- refractive indices of the FPR ($n_{s}$) and arrayed waveguides ($n_{c}$),
- waveguides width ($W_{x}$) and mode field radius ($w_{x}$).

The subscript *x* from $W_{x}$ and $w_{x}$ was replaced by *i* and *w* when referring to input/output waveguides or arrayed waveguides respectively. This set of parameters is known as physical parameters.

A second set of parameters was selected depending on the functionality needed for the device. This set is known as high level parameters, which are the basic requirements needed for the design of an AWG. These are:

- Number of channels ($N_{v}$): number of wavelengths to analyze
- Frequency channel spacing ($\Delta \nu_{ch}$): frequency difference between adjacent channels
- Channel bandwidth ($\Delta \nu_{bw}$): range of frequencies transmitted through each channel
- Loss non uniformity ($L_{u}$): amplitude signal relation between the central and most external output waveguide.

**Figure 2:**
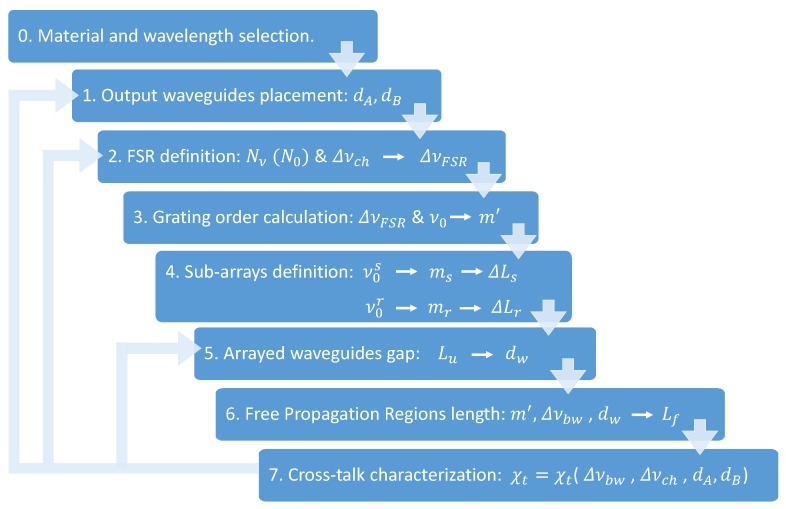
IOSS design flow. Step (0) selection of the material and wavelength to work with, depending on the application. Step (1) determination of output waveguides placement. Step (2) free spectral range (FSR) definition through the number of channels and the channel spacing selected. Step (3) AWG grating order calculation from FSR and central frequency. Step (4) sub-arrays determination: calculation of the central frequency and incremental length of each sub-array. Step (5) arrayed-waveguide gap determination through $L_{u}$ parameter. Step (6) free propagation regions (FPR) length calculation. Step (7) Crosstalk characterization from the device response.

In Figure 2, a flow chart for the design of the device is presented, and this section is structured accordingly.

### 3.1. Readout Scheme: Output Waveguides Distances

As introduced in the previous section, the IOSS output plane was divided into two zones of similar dimensions devised to host the output waveguides for the sensing and reference signals, shown in Figure 1a. Distinct read-outs systems can be used depending on the application. For example, for wavelength meter devices, 3 × 3 interferometers are employed to track the input device wavelength [25]. Although this is not the goal of the IOSS device, it can help us to acquire more accurate information by means of measuring multiple points at the output.

**Figure 3:**
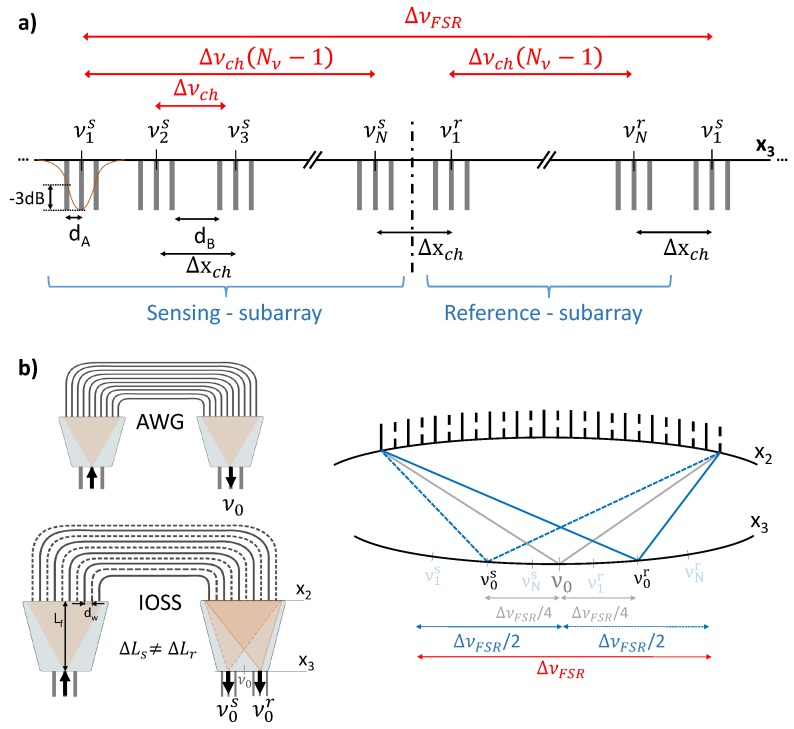
(**a**) Diagram of IOSS FSR response, channels and output ports location at output plane $x_{3}$. (**b**) Comparison between conventional AWG and an IOSS device in terms of central frequency.

Therefore, IOSS readout scheme presents two possible configurations depending on the transduction method employed; frequency shift analysis or absorption analysis. In the case of absorption analysis, the output power of the sensing channels was altered due to the absorption of the sample, hence just a single output waveguide per channel was needed to observe these changes. On the contrary, for refractrometry, the frequency shift was translated into a focal point displacement at $x_{3}$ plane, thus, it was necessary more than one output waveguide to track the focal point trajectory. Consequently, the output for frequency shift analysis consists of a set of three output waveguides for each of the sensing channels, as shown in Figure 3a. The gap between these waveguides ($d_{A}$), related to the waveguide cross-section and field decay, needed to be short enough to capture the frequency shift due to light-matter interaction in the sensing sub-array. In turn, the distance between external waveguides of adjacent channels ($d_{B}$) must be adequate to fulfill the target crosstalk specification for the application. The formulation presented sets these distances for a given amount of field decay as reference, but other criterion can be chosen. Hence, $d_{A}$ was set to the distance at which the central output signal reaches half of its maximum power (i.e, a power decay of −3 dB) due to frequency shift. The field amplitude profile of the signal at an output waveguide can be expressed as a normalized power Gaussian function:

(1) $$b_{i}\left(x_{3}\right)=\sqrt[{4}]{\frac{2}{\pi w_{i}^{2}}}e^{-{\left(\frac{x_{3}}{w_{i}}\right)}^{2}},$$

where $w_{i}$ is the mode field radius at the waveguides which varies according to the materials and the waveguide geometry of the technology platform selected. Using this expression, the aforementioned power ratio for adjacent waveguides in a given output channel is given by:

(2) $$d_{A}=\sqrt{\frac{\ln2}{2}}w_{i}.$$

Likewise, the distance $d_{B}$ is set by a power decay of −10 dB, with respect to the adjacent channel central waveguide. Again, from Equation (1):

(3) $$d_{B}=\sqrt{10^{\left(10/20\right)}}w_{i}.$$

The technology (materials) and lithography resolution (cross-section width, height and gaps) are key in this early part of the design, since they limit the minimum channel spacing for the device. By combining both distances, following Figure 3a, the separation between adjacent channels is:

(4) $$\Delta x_{ch}=2d_{A}+d_{B}.$$

It is important to highlight that when the analysis to be carried out does not involve frequency shift but only intensity fluctuations, a single output waveguide was enough, instead of three output waveguides per channel. For that case, $d_{A}$ = 0 in our formulation.

### 3.2. Readout Scheme: Optical Frequencies

Following the flow chart in Figure 2, the next step is to link the previously set distances to the optical frequency high level parameters set by the application.

Firstly, the number of channels, $N_{v}$, and the channel bandwidth, $\Delta \nu_{ch}$, were chosen according to the application. They were settled depending on the analyte under study, since the spectrum range to analyze may vary as well as wavelengths of interest. This is related to the spatial distance between adjacent channels ($\Delta x_{ch}$) previously set. Following the concept and illustration of Figure 1a and Figure 3a, two sets of $N_{v}$ frequency channels (sensing and reference) need to be allocated in the output coordinate. Hence, the free spectral range (FSR) of the device needs to be:

(5) $$\Delta \nu_{FSR}\geq 2\Delta \nu_{ch}N_{\nu }.$$

Next step on IOSS design was the calculation of the grating order ($m^{\prime }$). To obtain this parameter, the mathematical formulation was similar to a conventional AWG and can be examined in [14,24]. Thus, only the final result is shown here from which we can obtain $m^{\prime }$ value, since $\Delta \nu_{ch}$ and $\nu_{0}$ are set as inputs for the design. In order to take into account the waveguide dispersion, the modified grating order (*m*) is employed, in which the effective index and the group index are considered:

(6) $$\begin{array}{cc} m^{\prime } & =\frac{\nu_{0}}{\Delta \nu_{FSR,0}},\\ m & =\left\lfloor \frac{n_{c}}{n_{g}}m^{\prime }\right\rfloor . \end{array}$$

Finally, the optical frequencies for the sub-arrays are engineered. As explained in the concept section, the sub-arrays have to point to separate zones. Therefore, the sensing and reference sub-arrays must be devised to work at different central frequencies ($\nu_{0}^{s}$ and $\nu_{0}^{r}$ respectively, Figure 3b). In a regular AWG, $\nu_{0}$ was employed to set some physical dimensions, such as the array waveguide spacing ($d_{w}$) and the FPR length ($L_{f}$), dimensions that will be explained in detailed in the next subsection. But an IOSS device, with two sub-arrays with distinct central frequencies, can not have two different FPRs. Because of this, both sub-arrays were related to $\nu_{0}$ to be obtained later on the common values of $d_{w}$ and $L_{f}$. In the following equations, super/subscripts ‘s’ and ‘r’ stand for ‘sensing’ and ‘reference’ respectively. Departing from $\nu_{0}$ and Figure 3b, their respective central frequencies can be derived as:

(7) $$\begin{array}{c} \nu_{0}^{s}=\nu_{0}-\frac{\Delta \nu_{ch}N_{\nu }}{2}, \end{array}$$

(8) $$\begin{array}{c} \nu_{0}^{r}=\nu_{0}+\frac{\Delta \nu_{ch}N_{\nu }}{2}. \end{array}$$

From these equations, the grating order for each sub-array can be calculated as well through Equation (6) and therefore, the length increments are given by [24]:

(9) $$\begin{array}{c} \Delta L_{s}=\frac{c\;m_{s}}{n_{c}^{s}\;\nu_{0}^{s}}, \end{array}$$

(10) $$\begin{array}{c} \Delta L_{r}=\frac{c\;m_{r}}{n_{c}^{r}\;\nu_{0}^{r}}. \end{array}$$

### 3.3. Focusing and Periodicity: Arm Spacing and FPR Length

Once the sub-arrays were defined, the following steps of the flow design chart link $L_{u}$ and $\Delta \nu_{bw}$ high level parameters with the parameters calculated in the previous sections to obtain the physical dimensions $d_{w}$ and $L_{f}$ still unset. The formulation presented in this section is the same as that of a conventional AWG [14,24], for this reason only the most relevant equations are shown.

The far field from the waveguides in the array resulted in a non-uniform power distribution, with a maximum in the center, $x_{3}=0$, and decaying towards the sides. This is characterized by the loss non-uniformity parameter ($L_{u}$), which is settled by the designer taking into account a trade-off: $L_{u}$ lower values will provide a flat response between IOSS channels, but will produce high secondary lobes at the output signal. $L_{u}$ can be linked to the distance between arrayed waveguides ($d_{w}$) over the $x_{1}$ and $x_{2}$ planes (Figure 3b):

(11) $$d_{w}=\frac{w_{w}\;\pi \;m\;\Delta \nu_{ch}\;N_{\nu }}{\nu_{0}}{\left[ln\left(10^{\frac{L_{u}\left(dB\right)}{20}}\right)\right]}^{-\frac{1}{2}}.$$

On the other hand, the channel bandwidth ($\Delta \nu_{bw}$) can be fixed as input in the design flow. It is defined as the full width at half maximum of the output port passband and it takes into account the frequency spatial dispersion (γ), which relates the displacement of the focal points along plane $x_{3}$ with the change in frequency. From the desired $\Delta \nu_{bw}$, the length of the FPR ($L_{f}$) can be obtained:

(12) $$\Delta \nu_{bw}=2\gamma w_{i}\sqrt{2\;ln\left(10^{3/20}\right)}=2\frac{d_{w}\;\nu_{0}^{2}\;n_{s}}{m\;c\;L_{f}}w_{i}\sqrt{2\;ln\left(10^{3/20}\right)},$$

where $n_{s}$ is the refractive index of the FPR. Note that in this derivation, the central frequency $\nu_{0}$ and its grating order $m^{\prime }$ are employed, as in a regular AWG, to set $d_{w}$ and $L_{f}$, whereas the IOSS consists of two arrays with different central frequencies and grating orders. This is a compromise approximation to keep the arrays (not perfectly) focused using a regular Rowland mounting for the device layout, but further arbitrary mountings could be devised to optimize the focusing of each sub-array independently [26].

Once $d_{w}$ and $L_{f}$ are set, the far field from the input waveguide is used to calculate the number of waveguide in the array, *N*. For this, the amount of optical power collected by the array has been previously settled by the designer.

All the above resulted in the IOSS spectral response, that at some point and due to some limitations (e.g., the aforementioned lithographic resolution, among others), may not fulfill all the high-level parameters used as inputs. In this case, the design can be iterated, and this is highlighted for steps 1, 2 and/or 5 in the design flow of Figure 2.

## 4. Reference Designs

In this section, two reference designs are presented working at the visible (VIS) and the near infra-red (NIR) wavelength ranges respectively. Both correspond to an IOSS device devised as a self-referenced absorption spectrometer. This configuration directly involved two design considerations: (i) a single output waveguide was needed per channel as only intensity fluctuations will be analyzed, and (ii) all sensing windows must have had the same length to avoid an extra phase change coming from the sensing sub-array, as it was explained in Section 2. Furthermore, the Beer–Lambert law can be then applied directly to the results obtained in order to procure information on the concentration of the sample.

Firstly, the VIS device design is presented for a particular application in the field of food quality control to determine the concentration of a known dye in aqueous solution. Secondly, a NIR device design was elaborated and analyzed for an application in the quality control in alcoholic beverages field. For both cases, the material system chosen is silicon nitride on silica (Si${}_{3}$N${}_{4}$/SiO${}_{2}$) [4], since this technology has been traditionally employed in evanescent field sensing from VIS to NIR [7].

The most compact configuration has been settled for both designs, which means $\Delta \nu_{FSR}=2\Delta \nu_{ch}N_{\nu }$. This implies that the channels of the two sub-arrays are periodically interleaved, with a spectral separation of $\Delta \nu_{ch}$ between sensing and reference channel sets.

The first simulation step is to obtain the propagating mode field and the effective refractive indexes of all the different cross-sections presented in the design (straight, bend and sensing waveguides, as well as slab coupler and taper waveguides) employing a mode solver software. For the second step, the layout of the device was built following the design flow presented in Section 3. Once the IOSS was built, the device parameters (number of arrayed waveguides and their length, $L_{f}$ $w_{x}$, $n_{s}$, $n_{c}$, etc.) were exported to simulate the sensor response in Matlab. As mentioned in Section 3.1, these simulations were based on Fourier optics, applying paraxial approximation by using Gaussian modes fields as it was done in [14,19]. A Gaussian function was employed to fit the actual field in the waveguides core, with the Gaussian width as fitted by the mode solver. The field at the image plane of the FPR was obtained through Fresnel diffraction of the Gaussian function, and the overlap integral was calculated to acquire the field at the arrayed waveguides. The light propagation in the arrayed waveguides was simulated by regular complex exponential terms, to account for the phase change over the waveguide lengths. Once again, the Fresnel diffraction was employed to obtain the response of the device at plane $x_{3}$.

### 4.1. Visible Wavelength Range Device

The design presented in this section corresponds to the application of the IOSS device in determining the concentration of a coloring dye, sunset yellow (SY), of use in the food industry [27], among others. These dyes are known to be innocuous below certain concentration levels [28]. For the particular case of the SY in a water solution, the absorption spectra is well known at a given concentration [29], having significant spectral components in the visible range, with relevant peaks in between 470 and 520 nm. Therefore, the nominal design wavelength is settled to $\lambda_{0}$ = 495 nm. We depart then from a Si${}_{3}$N${}_{4}$ technology with substrate height buried oxide (BOX) 2 μm, with a waveguide cross-section WxH of 400 nm × 100 nm (with bends of radius 35 μm), so as to ensure single-mode and single polarization (TE) in the targeted spectral range. The upper cladding oxide height was 1.5 μm. The effective refractive indexes obtained from the simulation were: $n_{c}$ (λ = 495 nm) = 1.6582, $n_{g}$ (λ = 495 nm) = 2.1114, and $n_{s}$ (λ = 495 nm) = 1.9976. The channel wavelength spacing was set to ($\Delta \lambda_{ch}$ = 2 nm) resulting in a $\Delta \nu_{FSR}$ = 19.57 THz and a diffraction order *m* = 24. The values for the IOSS arm spacing ($d_{w}$ = 1.2 μm) and the channel bandwidth ($\Delta \nu_{bw}$ = 0.2$\Delta \nu_{ch}$) have been engineered to minimize the crosstalk. According to the design equations, a slab length $L_{f}$ = 95.91 μm was obtained.

The number of wavelengths to be analyzed was four ($\lambda_{1}\approx$ 476 nm, $\lambda_{2}\approx$ 487 nm, $\lambda_{3}\approx$ 502 nm and $\lambda_{4}\approx$ 515 nm). These wavelengths have been selected to sample the decay of the SY absorption peak (Figure 2 from [29]). This resulted in eight channels, corresponding to four couples of reference and sensing channels, as described in Section 2 and Section 3. Note that the design was set to have a channel wavelength spacing of 2 nm, but the four targeted wavelengths can still be analyzed owing to the spectral periodicity of these devices.

The length of the openings in the sensing arms was set to 200 μm. This was determined in light of the different absorption levels of the target analyte [29] and the amount of evanescent field in the waveguide cross-section propagated mode employed (18%), for the different concentrations of the analyte to provide an observable absorption in the spectra recorded with the device. For the simulation, the SY concentration and bulk absorption were taken from Figure 2 of reference [29].

**Figure 4:**
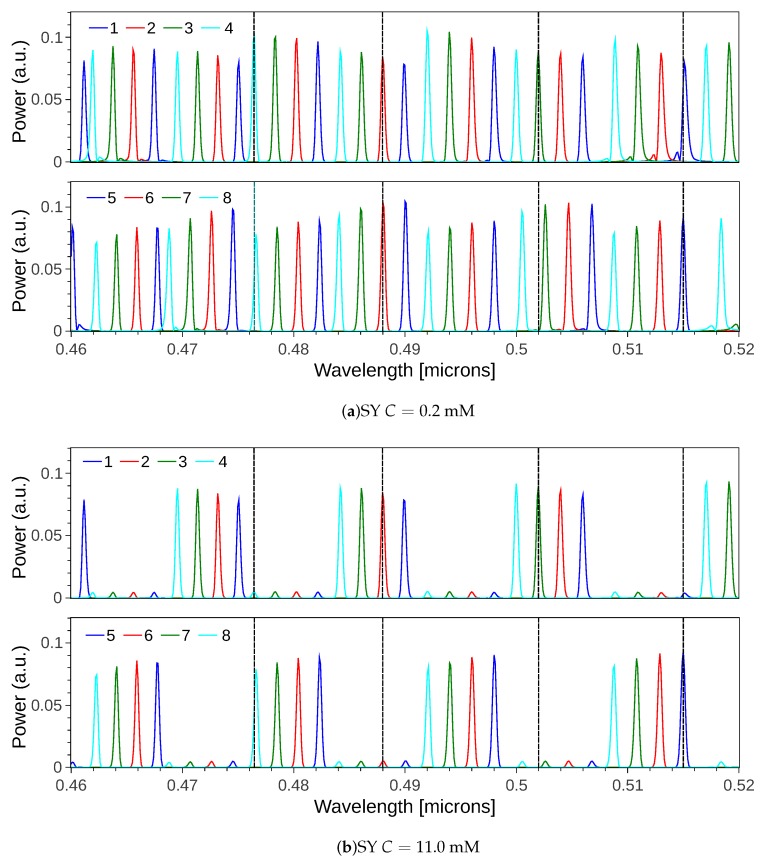
IOSS spectral response for channels 1–8 for sunset yellow (SY) concentrations (**a**) *C* = 0.2 mM and (**b**) *C* = 11.0 mM. Channels are colored in pairs, with same color for those having spectrally aligned responses. The dark dashed vertical lines correspond to the targeted wavelengths 476, 487, 502 and 515 nm.

The calculated spectral responses for the eight channels are represented in Figure 4, where graphs (a) and (b) show the sensing and reference contributions of each channel (1–4 and 5–8) for lower (a) and higher (b) SY concentrations respectively. The eight channels are represented in two separated groups to easily observe the duplication of the transmitted wavelength between channels 1–5, 2–6, 3–7 and 4–8. The simulation was done assuming a flat spectral broadband signal as input, for illustration purposes. Despite this, as mentioned, the operation of this design was to be done with narrow band light sources, i.e., with spectral content around the wavelengths of interest, with ideally no overlap with the adjacent channels (this is 2 nm in the present example). By comparing panels (a) and (b), the presence of absorption reflects the origin of the different spectral contributions in each channel. In other words, despite the fact that one may tend to think from the device concept that each output solely receives contributions from either the reference or the sensing sub-array, the simulation showed how this was wavelength dependent. To be precise, e.g., for λ= 487 nm, channel 2 kept a high power level, whereas the power of its twin channel 6 was reduced due to absorption in the sensing sub-array. However, for λ= 476 nm, whereas the channel 4 power level was affected by absorption, its mated channel number 8 was not. As a conclusion, in the case the wavelengths under analysis fall within one FSR of the device, one can strictly refer to channels 1–$N_{o}$/2 as reference/sensing channels, and sensing/reference to the other half. On the contrary, when the targeted wavelengths are an odd number of FSRs away from each other, this changes as illustrated. Nonetheless, this did only have impact on the calculation of the power ratio $P_{s}/P_{r}$, for which the proper readout signal needs to be assigned to each. It should be noted that sensing and reference channels were not evenly spaced at plane $x_{3}$. This is due to the fact that none of the sub-arrays had its optimum $L_{f}$, but an average length which was settled to cover both of them, as it was already mentioned in Section 3.3.

**Figure 5:**
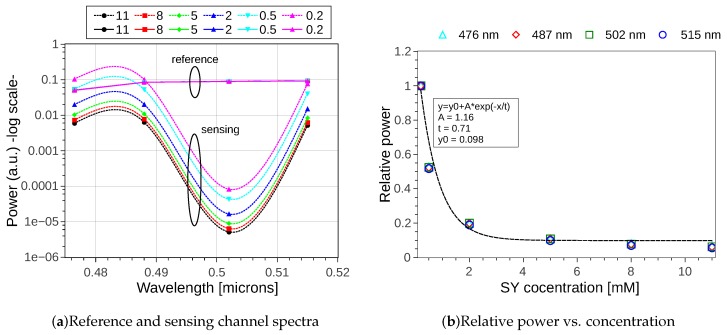
(**a**) Reference and sensing channel spectra for the targeted wavelengths 476, 487, 502 and 515 nm, with reference channels (solid lines) and sensing channels (dashed lines) for different SY concentrations. The values are interpolated using smoothed (spline) lines. (**b**) Relative power change with respect of the starting concentration (*C* = 0.2 mM) for the four targeted wavelengths.

For a set of concentrations in the range [0.2,11.0] mM, the spectral response for the targeted wavelengths was calculated, and the results are given in Figure 5. The power in arbitrary units, obtained for these wavelengths, is plotted in Figure 5a, for both the reference and sensing readout channels, in solid and dashed lines respectively. As expected, the power in the reference channels did not vary with concentration, since the reference arms are not in contact with the analyte. Conversely, the power in the sensing channels changed accordingly to the absorption in the analyte, which in our present example was increased when the SY concentration was increased. Note the obtained attenuation spectra corresponded to the aforementioned bulk absorption spectra of Figure 2 from reference [29]. By taking as reference the relative power between the sensing and reference readout channel for C=0.2 mM, the decaying evolution of this power ratio for increased concentrations is plotted in Figure 5b, for the four targeted wavelengths. As it can be appreciated, the variation of the sample concentration was similar for the four analyzed wavelengths. Hence, by operating at a well-known wavelength and starting concentration value, with an initial device calibration, the analyte concentration can be determined.

### 4.2. Near Infrared Device

As an example of an IOSS device working in the NIR wavelength range, an absorbance sensor has been designed to measure ethanol concentration in solution. One application can be found in the food quality control field for the alcohol determination in beverages [30]. Data from [31] have been taken in which several samples of ethanol-water mixtures at different ethanol concentrations are analyzed. Hence, this study was focused on the ethanol concentration measured around 1450 nm where the ethanol–water mixture spectrum shows an absorption peak, thus this will be the nominal designed wavelength ($\lambda_{0}$) of our device. The NIR design of the IOSS was developed in a similar way to the previous section. The substrate height in this case is 3 μm while maintaining the cladding oxide height to 1.5 μm. The waveguide cross-section needed to be 800 nm × 300 nm (W × H) to keep the single-mode and single polarization (TE) configuration, and bend radius of 75 μm were used. The propagation indexes obtained for this simulation were: $n_{c}$ (λ = 1450 nm) = 1.5702, $n_{g}$ (λ = 1450 nm) = 1.9314, and $n_{s}$ (λ = 1450 nm) = 1.6757. As for the VIS configuration of IOSS, four target wavelengths were analyzed centered at 1435, 1443, 1451 and 1459 nm (approximated values) resulting in eight channels (four sensing channels plus four reference channels). The channel wavelength spacing was set to 1.6 nm resulting in a $\Delta \lambda_{FSR}$ = 1.824 THz, thus, the diffraction order *m* = 92. Finally, the arm spacing was engineered to be $d_{w}$ = 3 μm, providing the slab length $L_{f}$ = 158.47 μm.

**Figure 6:**
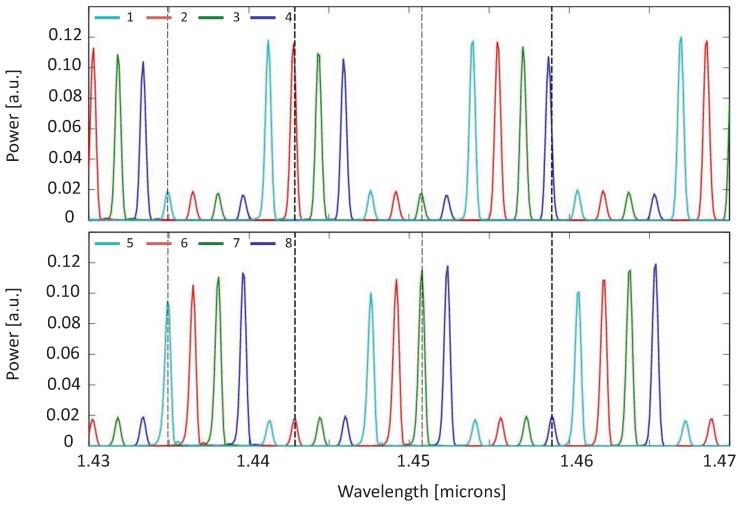
IOSS spectral response for channels 1–8 for ethanol concentrations (a) C=20 wt %. Channels are colored in pairs, with same color for those having spectrally aligned responses. The dark dashed vertical lines correspond to the targeted wavelengths 1435, 1443, 1451 and 1459 nm.

**Figure 7:**
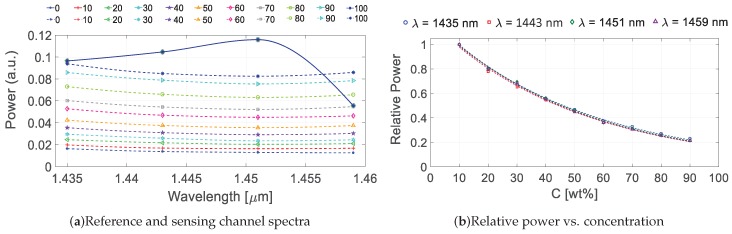
(**a**) Reference and sensing channel spectra for the targeted wavelengths 1435, 1443, 1451 and 1459 nm, with reference channels (solid lines) and sensing channels (dashed lines) for different ethanol concentrations [0–100 wt %]. The values are interpolated using smoothed lines. (**b**) Relative power change with respect of the starting concentration (*C* = 10 wt %) for the four targeted wavelengths.

Since the IOSS operation is analogous for NIR and VIS, the spectral results are very similar to the ones obtained in Figure 4. So in order to avoid repetition, only one chart for IOSS-NIR response at low ethanol concentration is shown (Figure 6). Note that the concentration in this example is given in mass fraction (wt%). Following the same procedure as explained in Section 4, the absorption spectrum of different ethanol concentration samples can be recovered. Figure 7a shows the power in arbitrary units obtained at the sensing (dashed lines) and reference (continuous lines) channels for ethanol concentrations between 0–10 wt % in aqueous solution. Once again, the output power of the references channels was kept constant since there was no interaction with the sample, while for sensing channels the power increased as the concentration did (reduced water content, hence lower absorption in the NIR). In Figure 7b the relative power ratio for the four wavelengths is represented, taking as reference the sensing and reference ratio at C = 10 wt %. In this plot, the values for C = 0 wt % and C = 100 wt % have been obviated since they correspond to pure water and pure ethanol respectively.

**Figure 8:**
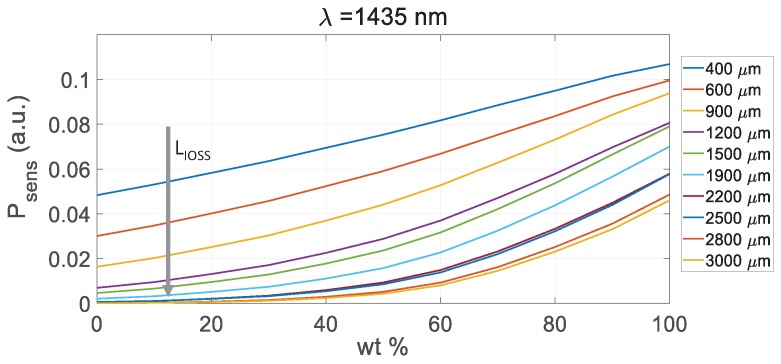
IOSS output power at the sensing channel λ=1435 nm for sensing path lengths from 400 μm to 3000 μm. The arrow shows the incremental trend of the sensing path.

The power variation between different concentrations is not so obvious as in the previous example (Figure 5a), hence further optimization of the device can be performed regarding the sensing path length. A simulation for the four targeted wavelengths was performed by sweeping the sensing path length from 400 μm (current value) to 3000 μm. The results are shown in Figure 8 for λ = 1435 nm (similar results for the rest of wavelengths, so only these are shown) where it can be appreciated that for longer sensing paths the output power was almost null when analyzing low concentrations. The ideal sensing path length for this application would then be 900 μm, since the power at lower concentrations was still considerable. In addition, it presents the higher slope which provides the greater difference between concentrations, and thus, affords better sensitivity to the sensor.

## 5. Conclusions

A novel spectroscopic sensor structure based on a modified AWG has been presented in this article. The formulation and design modelling follow a similar procedure as an ordinary AWG, with the particularity of the sub-array definition. This sub-array division allow the sensing paths to be defined within the same device, resulting into reduced footprint when compared to parallel architectures with demultiplexing and sensing in a single device. The use of this proposed device to different applications is possible by selecting a proper combination of integrated technology, detectors and light sources. Future works will address the formulation of the device to be used as spectroscopic refractive index change sensor.

## 6. Patents

There is a patent resulting from the work reported in this manuscript. P. Muñoz, B. Gargallo, G. Micó, D. Pastor (2018). PCT/ES2017/070794. Spain: Oficina Espaõla de patentes y marcas (OEPM).
